# Radiological Assessment and Outcome of Local Disease Progression after Neoadjuvant Chemotherapy in Children and Adolescents with Localized Osteosarcoma

**DOI:** 10.3390/jcm9124070

**Published:** 2020-12-17

**Authors:** Adriana Fonseca, Anne L. Ryan, Paul Gibson, Eleanor Hendershot, Sevan Hopyan, Marilyn Ranson, Jennifer Stimec, Abha A. Gupta

**Affiliations:** 1 Division of Hematology/Oncology, Hospital for Sick Children, Toronto, ON M5G 1X8, Canada; abha.gupta@sickkids.ca; 2 Department of Heamatology and Oncology, Perth Children’s Hospital, Perth 6009, Australia; anne.ryan@health.wa.gov.au; 3 Division of Hematology/Oncology, McMaster Children’s Hospital, Hamilton, ON L8N 3Z5, Canada; paul.gibson@mcmaster.ca (P.G.); hendershel@hhsc.ca (E.H.); 4 Department of Orthopedic Surgery, Hospital for Sick Children, Toronto, ON M5G 1X8, Canada; sevan.hopyan@sickkids.ca; 5 Department of Diagnostic Imaging, Hospital for Sick Children, Toronto, ON M5G 1X8, Canada; marilynranson@rogers.com (M.R.); jennifer.stimec@sickkids.ca (J.S.)

**Keywords:** osteosarcoma, MRI, radiological progression

## Abstract

Objective: We examined the interobserver reliability of local progressive disease (L-PD) determination using two major radiological response evaluation criteria systems (Response evaluation Criteria in Solid Tumors (RECIST) and the European and American Osteosarcoma Study (EURAMOS)) in patients diagnosed with localized osteosarcoma (OS). Additionally, we describe the outcomes of patients determined to experience L-PD. Materials and Methods: Forty-seven patients diagnosed with localized OS between 2000 and 2012 at our institution were identified. Paired magnetic resonance imaging of the primary tumor from diagnosis and post-neoadjuvant chemotherapy were blindly assessed by two experienced radiologists and determined L-PD as per RECIST and EURAMOS radiological criteria. Interobserver reliability was measured using the kappa statistic (κ). The Kaplan Meier method and log-rank test was used to assess differences between groups. Results: Of 47 patients (median age at diagnosis 12.9 years), 16 (34%) had L-PD (by RECIST or EURAMOS radiological definition). There was less agreement between the radiologists using EURAMOS radiological criteria for L-PD (80.9%, κ = 0.48) than with RECIST criteria (97.9%, κ = 0.87). Patients with radiologically defined L-PD had a 5-year progression-free survival (PFS) of 55.6%, compared to a 5 year-PFS of 82.7% in the group of patients without L-PD (*n* = 31) (Log rank *p* = 0.0185). Conclusions: The interobserver reliability of L-PD determination is higher using RECIST than EURAMOS. RECIST can be considered for response assessment in OS clinical trials. The presence of L-PD was associated with worse outcomes.

## 1. Introduction

Osteosarcoma (OS) is the most common malignant bone tumor of childhood [1]. The current standard therapeutic strategy involves the use of two cycles of neoadjuvant chemotherapy with high dose methotrexate, cisplatin and doxorubicin (MAP), followed by complete surgical resection of the primary tumor, and concluding with four more cycles of MAP for a total of 6 cycles (~28 weeks of chemotherapy) [2,3,4]. Despite intensive treatment, almost one third of patients with localized OS relapse within 2 years of diagnosis, most commonly with metastatic disease in the lungs [5].

The objective assessment of response to chemotherapy has been standardized using the Response Evaluation Criteria in Solid Tumors (RECIST), which defines progressive disease (PD) as a 20% increase in the sum of 2 diameters of a target lesion, taking as reference the smallest sum on study. In addition, the sum must also demonstrate an absolute increase of at least 5 mm [6]. It has been established as the imaging response guideline applied broadly to solid tumors protocols and clinical trials. The European and American Osteosarcoma Study (EURAMOS) trial was a large, multi-national clinical trial in osteosarcoma that used its own radiological assessment guidelines and instead defined PD as a 20% increase in any single dimension of the primary tumor when assessed radiologically in association with clinical features of progression such as recurrence of pain, swelling and/or serum alkaline phosphatase [7].

Patients who experience local progressive disease (L-PD) during neo-adjuvant chemotherapy pose a significant challenge for clinicians. Due to the lack of effective therapeutic approaches, the management of these patients is comprised of the continuation of similar chemotherapy regimens, removal from treatment protocol where applicable, and/or the addition of investigational new agents. Unfortunately, the outcome of these patients is suspected to be poor but has never been clearly documented [8].

In this study, we examined the interobserver reliability of local progressive disease (L-PD) determination using RECIST and EURAMOS in patients diagnosed with localized osteosarcoma (OS). Additionally, we describe the outcomes of patients who experienced L-PD.

## 2. Experimental Section

### 2.1. Patient Selection

**Figure 1:**
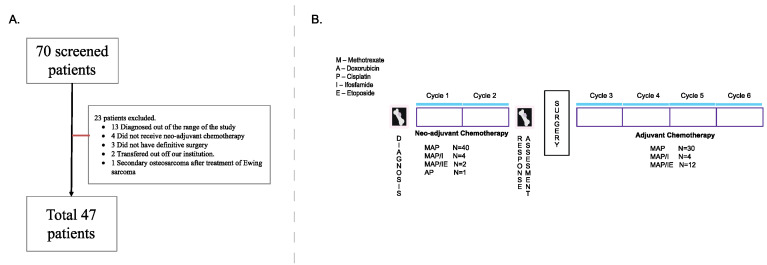
(**A**) Flow of patients during the study. (**B**) General treatment schema.

Following ethics approval by the institutional review board, pediatric patients (0–18 years) diagnosed with localized OS at our institution between January 2000 and December 2012 were identified through the institutional oncology database. Patients were eligible for review based on the following inclusion criteria: (1) localized (non-metastatic) disease at diagnosis, (2) high-grade OS, (3) diagnostic magnetic resonance imaging (MRI) of the primary lesion performed within 4 weeks prior to neo-adjuvant chemotherapy and (4) MRI following neo-adjuvant chemotherapy within 4 weeks prior to definitive resection. Patients who did not receive neoadjuvant chemotherapy or where clinical information was not available, were excluded. Patient flow during the study is represented in Figure 1A. A retrospective chart review was performed and patient demographics, disease characteristics, treatment information and outcomes were collected.

### 2.2. Radiological Review

Two expert musculoskeletal staff radiologists (**R1 and **R2), blinded to patient outcome, independently reviewed paired MRI scans of the primary tumor from diagnosis and post-neoadjuvant chemotherapy and measured the tumor components as per RECIST and EURAMOS radiological criteria. The following measurements were obtained: tumor length (TL: the greatest longitudinal distance of the lesion on sagittal or coronal imaging), tumor width (TW: horizontal extension, measured in axial images) and tumor depth (TD: anteroposterior extension from axial images). L-PD as per RECIST criteria (defined as ≥20% increase in the sum of diameters of the primary tumor, and in addition to the relative increase of 20%, the sum must also demonstrate an absolute increase of at least 5 mm) and the L-PD EURAMOS radiological criteria (defined as ≥20% in any dimension of the primary tumor) were calculated by an independent investigator. Patients were classified as having L-PD or not by both RECIST and EURAMOS.

### 2.3. Statistical Analysis

Concordance between radiologists rating L-PD using RECIST and EURAMOS criteria was assessed by Cohen’s kappa statistic (κ) [9]. The kappa statistics usually lie between 0 (absence of agreement) and 1 (absolute agreement). K were interpreted in a qualitative manner [10]. Progression free survival (PFS), was defined as the time lapsed from diagnosis to recurrence, progression or death of the disease. Overall survival was calculated from the time of diagnosis to death of any cause. The patterns of PFS were estimated using the Kaplan–Meier method [11]. The log-rank test was used to compare the group of patients with L-PD versus the group with no L-PD for EURAMOS and RECIST. All statistical analyses were performed using STATA statistical software (version 14.2; StataCorp LLC, College Station, TX, USA). As this was a retrospective study, no sample size calculations were performed. The number of patients included represents a convenience sample of all patients at the institution who met the eligibility criteria.

## 3. Results

### 3.1. Patient Characteristics

Of 70 patients identified, 47 met eligibility criteria (Figure 1A; the general schema of the treatment for these patients is seen in Figure 1B). Median age at diagnosis was 12.9 years (range 4.8–16.6). Table 1 describes demographics, tumor and treatment details for all evaluated patients. Primary sites of disease were similar to previously described cohorts of OS [7,12], with the majority (*n* = 40, 85%) occurring in the lower extremity. Sixteen patients (34%) had L-PD following neo-adjuvant chemotherapy using either RECIST or EURAMOS radiological definition by either radiologist. Chemotherapy was changed postoperatively in 10 patients: in 9/10 patients due to poor pathological response, while one patient had good pathological necrosis but pre-operative evidence of significant tumor growth. At the time of this study, the recommendation was to change chemotherapy based on pathological necrosis at the time of surgery.

Twenty-nine (61.7%) patients underwent limb salvage surgery with endoprosthesis. Twelve (25.5%) patients underwent amputation; three had below-knee amputation. Furthermore, vascular complications were observed in three patients initially planning to have endoprosthesis (*n* = 2) or rotationplasty (*n* = 1).

Twenty-five patients had good necrosis (>90%) and 22 had poor necrosis (<90%) in the entire cohort as depicted in Table 1. Out of the 22 patients with poor pathological necrosis, 4/22 (18%) were determined to have L-PD as per RECIST guidelines and 11/22 (50%) were determined to have L-PD as per EURAMOS guidelines.

### 3.2. Radiologist Agreement

There was moderate agreement between radiologists using EURAMOS radiological criteria for L-PD (κ 0.48 SE_k_ ± 0.13 (80.9%)), as compared to almost perfect using the RECIST criteria (κ 0.87 SE_k_ ± 0.14 (97.9%)), detailed in Table 2. When reporting evidence of radiological necrosis in the primary tumor there was slight congruency between radiologists with κ 0.33 SE_k_ ± 0.08, and agreement was calculated at only 52.4.8%.

### 3.3. Outcome after L-PD

**Figure 2:**
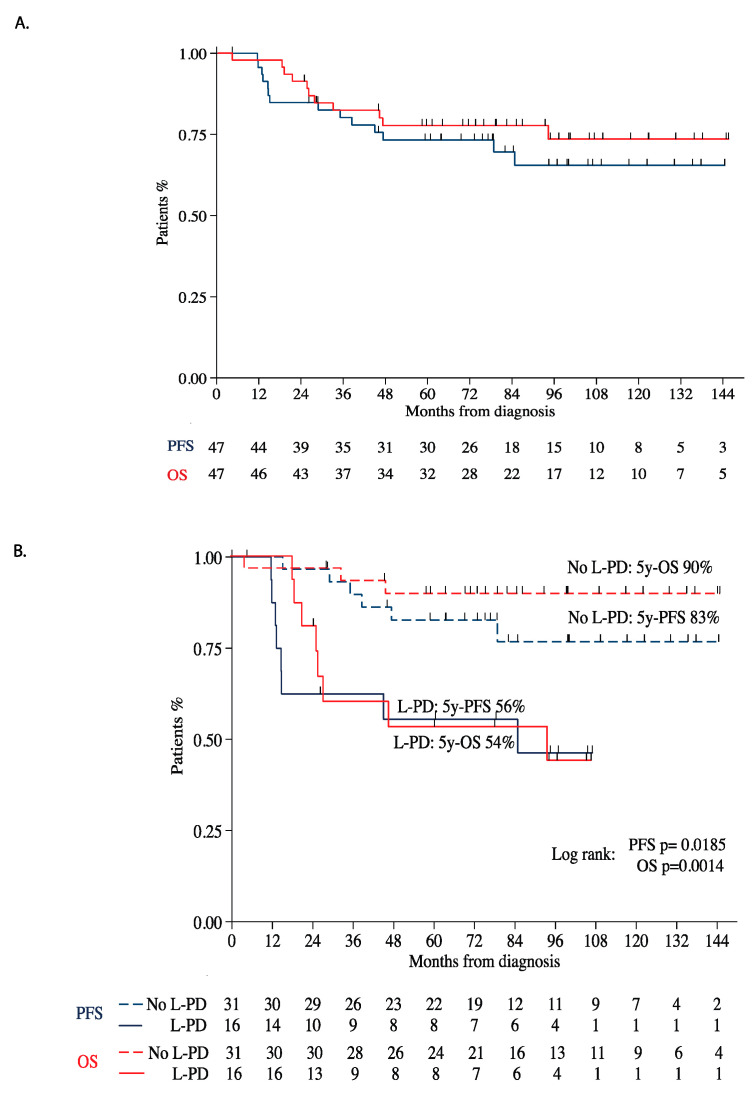
(**A**) Progression-free (PFS) and overall survival (OS) of the entire cohort; (**B**) PFS and OS of patients with and without local progressive disease (L-PD).

At a median follow-up of 6.5 years (0.3–17.3), a total of 14 (29.8%) events were observed in the entire cohort, all as distant relapses. The 5-year PFS and overall survival was 73.2% (95% CI 0.57, 0.83) and 78.1% (95% CI 0.63, 0.87), respectively (Figure 2A).

There were 8 (50%) events in sixteen patients determined to have L-PD by either criteria with a 5-year PFS was 55.6% (95% CI 0.28, 0.75). In contrast, there were 6 (19%) events in thirty-one patients without L-PD with a 5 year-PFS of 82.7% (95% CI 0.63, 0.9) (Log rank *p* = 0.0185) Figure 2B.

## 4. Discussion

RECIST criteria is the gold-standard for treatment response assessment in oncology clinical trials [6]; however, discrepancies in the evaluation of tumor response (including partial response, progressive disease and stable disease) between radiologists has been noted in many cancer types including bone and soft tissue sarcomas [13,14]. Osteosarcoma (OS) is a distinctive solid tumor with a substantial extracellular matrix that is often composed of calcified osteoid [15,16] which contributes to the clinical observation that OS uncommonly ‘shrinks’ or has a true partial response by RECIST criteria in response to chemotherapy. The EURAMOS team considered tumor volume, in additional to clinical findings, to perhaps represent a superior method of detecting treatment response in patients with OS. Our study highlights superior inter-observer reliability using RECIST criteria to identify radiological local disease progression compared with EURAMOS criteria. Importantly, our study highlights the need for centralized radiology review for tumor response assessment for multi-center clinical trials in OS.

Determining L-PD in patients undergoing treatment for OS has important clinical and therapeutic implications. One third of patients in this study were identified to have radiologically defined L-PD following standard MAP chemotherapy by either RECIST or EURAMOS assessment criteria. In our cohort, adjuvant chemotherapy with MAP-IE was given to 10/16 patients determined to have L-PD; the remaining 6 continued with MAP chemotherapy as per initial treatment plan. These observations reflect the difficulties clinicians face when managing patients who are deemed to have L-PD following neo-adjuvant chemotherapy, when no other effective therapeutic options exist. Our data suggest the outcomes of patients determined to have L-PD are associated with worse survival outcome; however, patients still had a prolonged survival with a 5-year PFS 55–60%. Prior studies have confirmed the association between primary tumor progression during neoadjuvant chemotherapy and poor outcomes [5,17,18,19]. A recent paper evaluating RECIST criteria suggested that in localized disease, L-PD was associated with a poor outcome, but that RECIST is a poor surrogate endpoint for survival in primary OS [8].

In our study, the surgical plan was not altered by the evidence of radiological L-PD. Surgical outcomes are another important consideration in the management of these patients. In the presence of L-PD, limb-preserving interventions maybe more difficult and could lead to more amputations [17].

This study is limited by its retrospective nature, which limited our ability to accurately capture real-time decision-making and clinical symptoms of tumor progression, which are an important component of EURAMOS. The inability to fully include the clinical criteria may explain the observed advantage when using RECIST vs. EURAMOS for response assessment. Additionally, the small sample size prevented us from including other significant clinical risk factors and therefore only the trend is described. Further, since the conduct of this study, MRI technology has continued to improve with the addition of dynamic contrast enhanced MRI and diffusion-weighted sequences [20,21]. These additional modalities may provide a more accurate assessment of tumor activity and L-PD.

## 5. Conclusions

In summary, inter-reader reliability to document L-PD was superior using the RECIST system compared with EURAMOS and can be considered for trials moving forward. Future trials of OS should consider centralized radiology review and maintain enrolment of those with L-PD to ensure follow-up and document disease outcome.

## Figures and Tables

**Table 1:** Patient characteristics.

**Patient Demographics**		***N* = 47**
	Age	
	Median (range) in years	12.9 (4.8–16.6)
	Gender	
	Male	20 (42.5%)
	Location	
	Lower extremity	40 (85.1%)
	Upper extremity	5 (10.6%)
	Other *	2 (4.26%)
**Treatment Information**	Type of surgery	
	Amputation	12 (25.5%)
	Endoprosthesis	29 (61.7%)
	Rotationplasty	6 (12.8%)
	Pathological Necrosis	
	<90%	22(46.81%)
	>90%	25(53.19%)
	Neoadjuvant Chemotherapy	
	MAP	40 (85.1%)
	Other (MAP/IE, MAP/I, AP)	7 (14.9%)
	Adjuvant Chemotherapy	*N* = 46 **
	MAP	30 (65.2%)
	Other	16 (34.8%)
	Chemo Changed postop	10 (21.7%)

Other: * 1 rib, 1 manubrium. ** One patient did not receive adjuvant chemotherapy. Abbreviations: MAP: methotrexate, doxorubicin, cisplatin; MAP/IE: methotrexate, doxorubicin, cisplatin/ifosfamide, etoposide.

**Table 2:** Interrater reliability between radiologists; rating progressive disease by Response evaluation Criteria in Solid Tumors (RECIST) and the European and American Osteosarcoma Study (EURAMOS).

RECIST				EURAMOS			
		R#2				R#2	
R#1		No L-PD	L-PD	R#1		No L-PD	L-PD
	No L-PD	42	1		No L-PD	31	2
	L-PD	0	4		L-PD	7	7
Interrater Agreement		Agreement	Kappa	Interrater Agreement		Agreement	Kappa
		97.9%	0.87			80.9%	0.48

R#1 = radiologist #1; R#2 = radiologist #2; L-PD: local-progressive disease.
